# Radio frequency hyperthermia system for skin tightening effect by filled waveguide aperture antenna with compact metamaterials

**DOI:** 10.3389/fbioe.2024.1378084

**Published:** 2024-03-28

**Authors:** Ikhwan Kim, Dong-Min Lee, Jae-Woo Shin, Gyoun-Jung Lee, Eun-Seong Kim, Nam-Young Kim

**Affiliations:** ^1^ RFIC Bio Center, Kwangwoon University, Seoul, Republic of Korea; ^2^ Department Electronic Engineering, Kwangwoon University, Seoul, Republic of Korea; ^3^ APR Device Center, Seoul, Republic of Korea

**Keywords:** RF hyperthermia, metamaterial, skin tightening, beauty device, waveguide aperture antenna

## Abstract

Radio frequency (RF) hyperthermia focuses on raising the target area temperature to a value exceeding 45°C. Collagen is stimulated when the temperature rises to 45°C at the dermal layer, resulting in skin tightening. However, most studies on RF hyperthermia have focused on tumor ablation or using electrodes to radiate an electromagnetic field, which is highly inefficient. This study proposed a non-invasive RF hyperthermia skin-tightening system with a compact metamaterial-filled waveguide aperture antenna. The proposed RF system increased the temperature by 11.6°C and 35.3°C with 20 and 80 W of 2.45 GHz RF power, respectively, within 60 s and exhibited a very focused effective area. Furthermore, a metamaterial was proposed to reduce the size of the waveguide aperture antenna and focus the electromagnetic field in the near-field region. The proposed metamaterial-filled waveguide aperture antenna was compact, measuring 10 mm × 17.4 mm, with a peak gain of 2.2 dB at 2.45 GHz. The measured hyperthermia performance indicated that the proposed RF system exhibited better power- and time-efficient hyperthermia performance than other RF hyperthermia systems in the cosmetic skin lifting commercial market. The proposed RF hyperthermia systems will be applied into a new generation of beauty cosmetic devices.

## 1 Introduction

**FIGURE 1:**
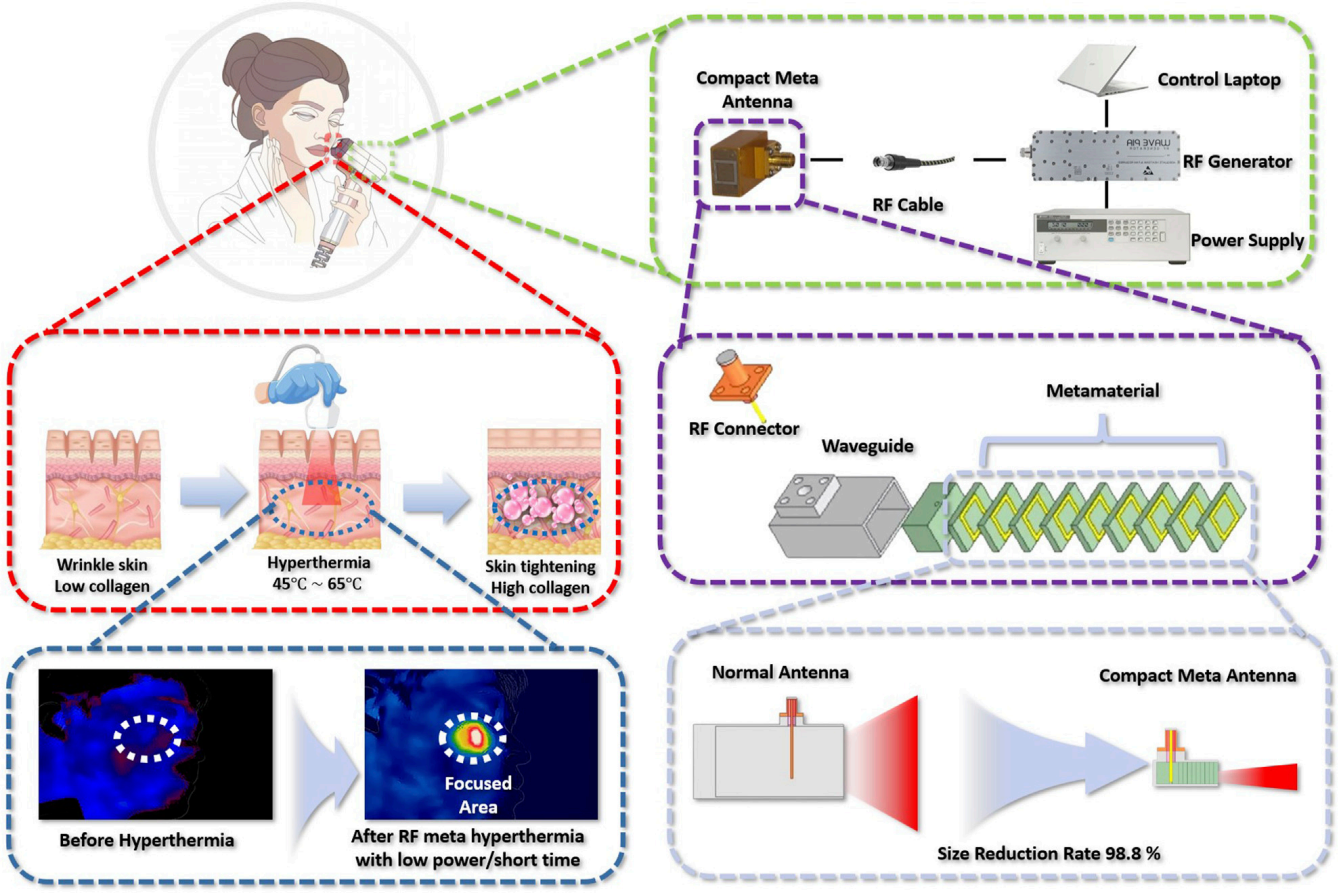
Hyperthermia for skin tightening therapy.

The beauty cosmetic home device market has grown tremendously. The Prescient & Strategic Intelligence report stated that the global home beauty device market was valued at USD 14,025.3 million in 2022 and is expected to reach USD 89,876.2 million by 2030. In the cosmetic field, it is widely reported that high temperatures between 45 and 65 ℃ can stimulate collagen production, leading to skin lifting and tightening (Yamamoto et al., 2006; Greene and Jeremy, 2014; Fabi, 2015; Jay, 2015). Skin tightening technology focuses on generating heat in the dermal layer, which is typically approximately 2–4 mm deep, as shown in Figure 1.

Microwaves and ultrasound are commonly used in skin-tightening hyperthermia therapy. Figure 1 shows microwave and ultrasound hyperthermia skin-tightening cosmetic machines.

Ultrasound treatment is well known for its safety and minimal side effects. However, it causes intense pain during the treatment (Shome, 2019; Lin, 2020). Furthermore, the effectiveness of skin-tightening hyperthermia therapy using ultrasound remains controversial. Additionally, the use of ultrasound gel is essential for treatment, which can be very uncomfortable (Malone et al., 2021).

Many studies have reported the effectiveness of using microwaves in skin-tightening therapy, which causes minimal discomfort. However, one of the disadvantages of using microwaves in skin-tightening hyperthermia therapy is the likelihood of burns owing to the high temperatures involved. To mitigate this risk, most therapies utilize a cooling tip or cooler to prevent skin burns while maintaining a high temperature beneath the skin surface (Weiss, 2013; Shin et al., 2019) [8–9]. Another drawback is its high cost and wide effective area. When microwaves are emitted, they spread widely, which renders focusing on the target area challenging and potentially increases the temperature in unintended areas.

Previous studies on radio frequency (RF) hyperthermia therapy have focused on cancer ablation using both invasive methods (Chiang et al., 2021; Kp and Arunachalam, 2022; Wang et al., 2022; Ganguly et al., 2023; Kp and Arunachalam, 2023) and noninvasive methods (Choi et al., 2014; Hajuagmadi et al., 2022; Mahmoud and Montaser, 2022). Most of these studies aimed at generating heat to ablate cancer cells. Because cancer cells have a higher relative permittivity than normal cells, they can heat more rapidly. Most of these studies employed phased-array antennas to focus the electromagnetic field on a target area, which is often quite small, to maximize the specific absorption rate (SAR). However, this approach typically requires considerable time to increase the temperature. For the convenience of cosmetic beauty device users, a rapid increase in temperature is desirable.

Previous studies on RF hyperthermia technologies for skin tightening employed electrodes to generate electromagnetic fields. Electrodes for electromagnetic-field generation are advantageous in the design of compact handheld devices. However, many of these designs do not consider impedance matching, which leads to impedance mismatch issues requiring higher power or longer treatment times to achieve the desired skin-tightening temperatures (Hurwitx and Darren, 2012; Weiss, 2013; Gongalsky et al., 2019; Prasad et al., 2019; Tanaka, 2019).

This study proposed a compact metamaterial-filled waveguide aperture antenna for cosmetic RF hyperthermia applications. The proposed antenna has a compact volume and small aperture size for a focused electromagnetic field in a specific target area and can endure high power. To the best of the author’s knowledge, this study proposes a metamaterial-filled waveguide aperture antenna for the first time and applies it to a cosmetic device for the first time, demonstrating high power efficiency.

## 2 Materials and methods

Among several antenna design considerations for RF cosmetic hyperthermia, the frequency band is the primary concern. Lower frequency bands, such as the 13.56 MHz band, can easily penetrate the skin, but they result in larger antenna sizes and difficulty in focusing the electromagnetic field in the near-field region. By contrast, mmWave bands facilitate compact antenna and RF system designs; however, they are typically more expensive than lower-frequency bands. Moreover, it is challenging for the mmWave signals to penetrate obstacles. To consider the fabrication costs and the penetration of the electromagnetic field, we chose the 2.45 GHz band, which is among the most used industrial, scientific, medical (ISM) bands and is more cost-efficient than mmWave bands.

The main design objective for hyperthermia antennas is to raise temperatures to a range of 45 – 65 ℃ . To efficiently deliver a strong electromagnetic field to a depth of 4 mm beneath the skin surface, the antenna should operate in the near-field region. Further, the antenna should be compact to facilitate focusing on small specific areas. If the antenna is too large, the electromagnetic field may extend to unwanted areas, potentially causing increased temperatures in these areas and resulting in side effects. In addition, the antenna should be capable of withstanding high powers. Power levels of up to 100 W may be required to generate heat as quickly as possible.

Although microstrip patch antennas are well known for their compact size and broadside beam patterns, they are not well suited for handling high power owing to the risk of dielectric breakdown caused by excessive heat between the radiator and the ground. To use a microstrip patch antenna at high power, a high-cost substrate must be used, which increases the fabrication cost. Waveguide antennas are well known for their durability. Typically, waveguide antennas are large, which makes focusing on the electromagnetic field in the near-field region challenging.

**FIGURE 2:**
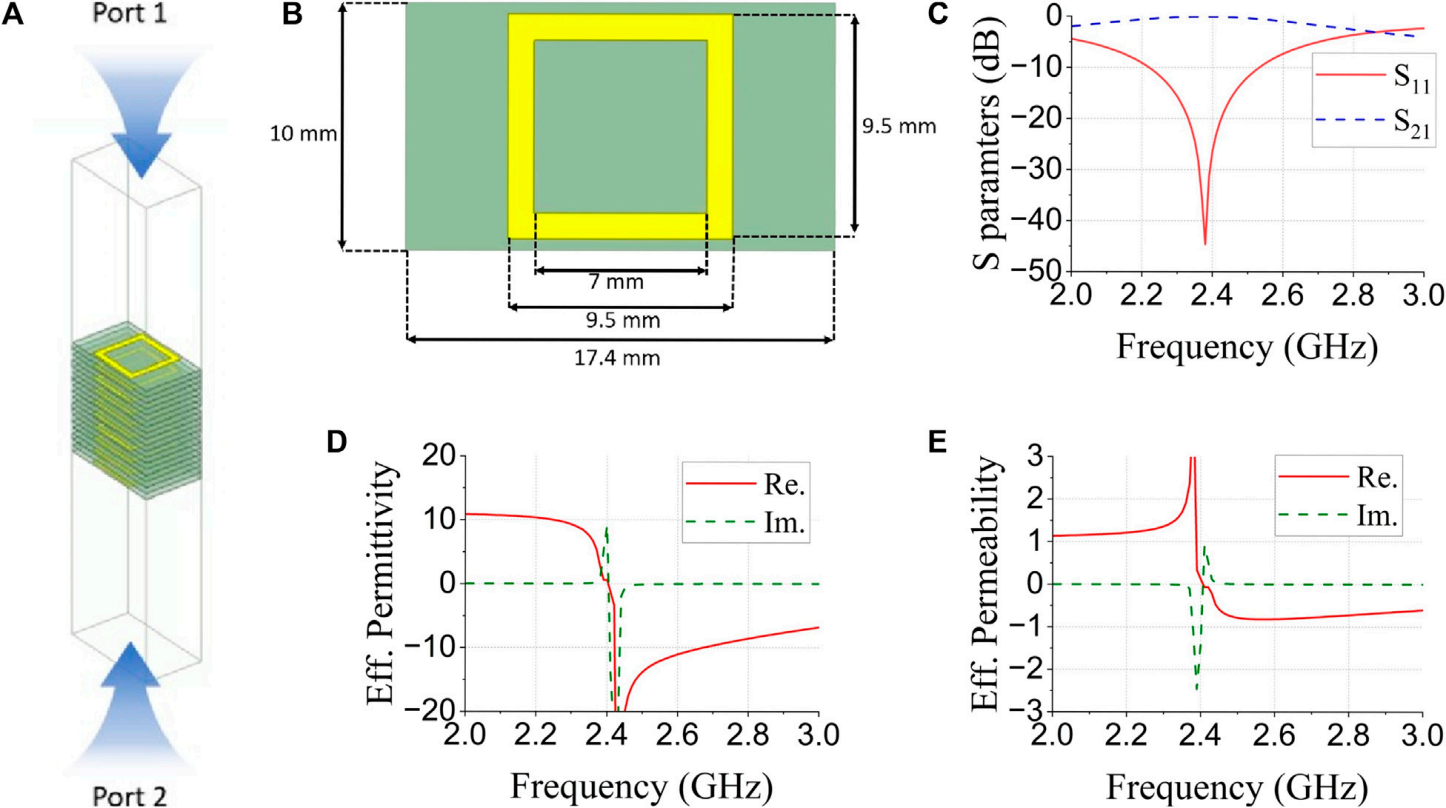
A shows the simulation setup for the proposed metamaterial. The effective permittivity and permeability of a metamaterial can be extracted from the metamaterial S parameters (Numan and Sharawi, 2013). The effective permittivity and permeability of the metamaterials were calculated using Eqs 1–4; where f is the frequency, c is the speed of light, and d is the largest dimension of the metamaterial. **(A)** metamaterial for minimizing size of waveguide. **(B)** specific values of the proposed metamaterial. **(C)** simulated S parameters of the proposed metamaterial, **(D)** effective permittivity. **(E)** effective permeability.

Metamaterials, also known as double-negative materials, exhibit negative permittivity and permeability in specific frequency bands. Several studies have demonstrated that metamaterials can enhance gain and minimize the size of an antenna (Pan et al., 2016; Yue et al., 2016; Zhu et al., 2019; Ikhwan et al., 2024). However, no studies have used metamaterials to minimize the waveguide size Figure 2. Proposed metamaterial for minimizing size of waveguide (a) metamaterial electromagnetic analyzed simulation setup and (b) specific view of metamaterial, (c) simulated S parameters of proposed metamaterial, (d) extracted effective permittivity of metamaterial, (e) extracted effective permeability of metamaterial

$$k_{0}=\frac{2\pi f}{c}$$ (1)

$$z=\pm \sqrt{\frac{{\left(1+S_{11}\right)}^{2}-{S_{21}}^{2}}{{\left(1-S_{11}\right)}^{2}-{S_{21}}^{2}}}$$ (2)

$$n=\frac{1}{k_{0}d}\left[I\left(\ln\left(\frac{S_{21}}{1-\frac{S_{11}\left(z-1\right)}{z+1}}\right)\right)-j\left(R\left(\ln\left(\frac{S_{21}}{1-\frac{S_{11}\left(z-1\right)}{z+1}}\right)\right)\right)\right]$$ (3)

$$\varepsilon_{eff}=\frac{n}{z}$$ (4)

$$\mu_{eff}=n\times z$$ (5)

Figure 2B shows the specific values of the proposed metamaterial. A rectangular pattern, made with copper, was printed on a Teflon substrate (thickness = 2 mm, $\varepsilon_{r}$ = 2.4), and nine Teflon substrates with a rectangular pattern were stacked.

Figure 2C shows the simulated S parameters of the proposed metamaterial, and (d) and (e) show the effective permittivity and permeability extracted from the simulated S parameters. The proposed metamaterial resonated at approximately 2.4 GHz and had negative permittivity and permeability around the resonant frequency. The proposed metamaterial was designed to minimize the size of the waveguide aperture antenna.

**FIGURE 3:**
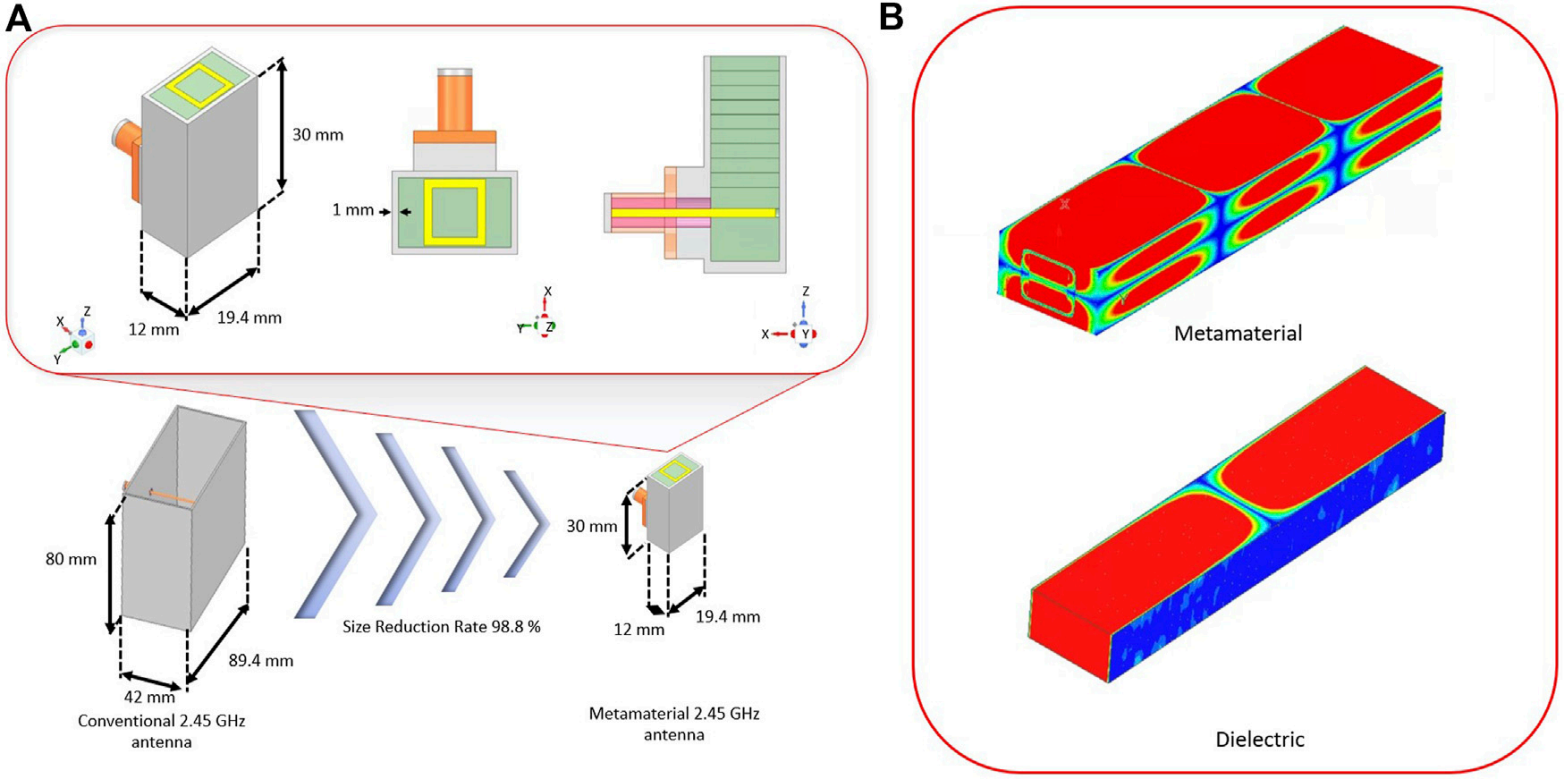
**(A)** Proposed compact metamaterial-filled waveguide aperture antenna and **(B)** electric field distribution of waveguide filled with proposed metamaterial and dielectric.

**FIGURE 4:**
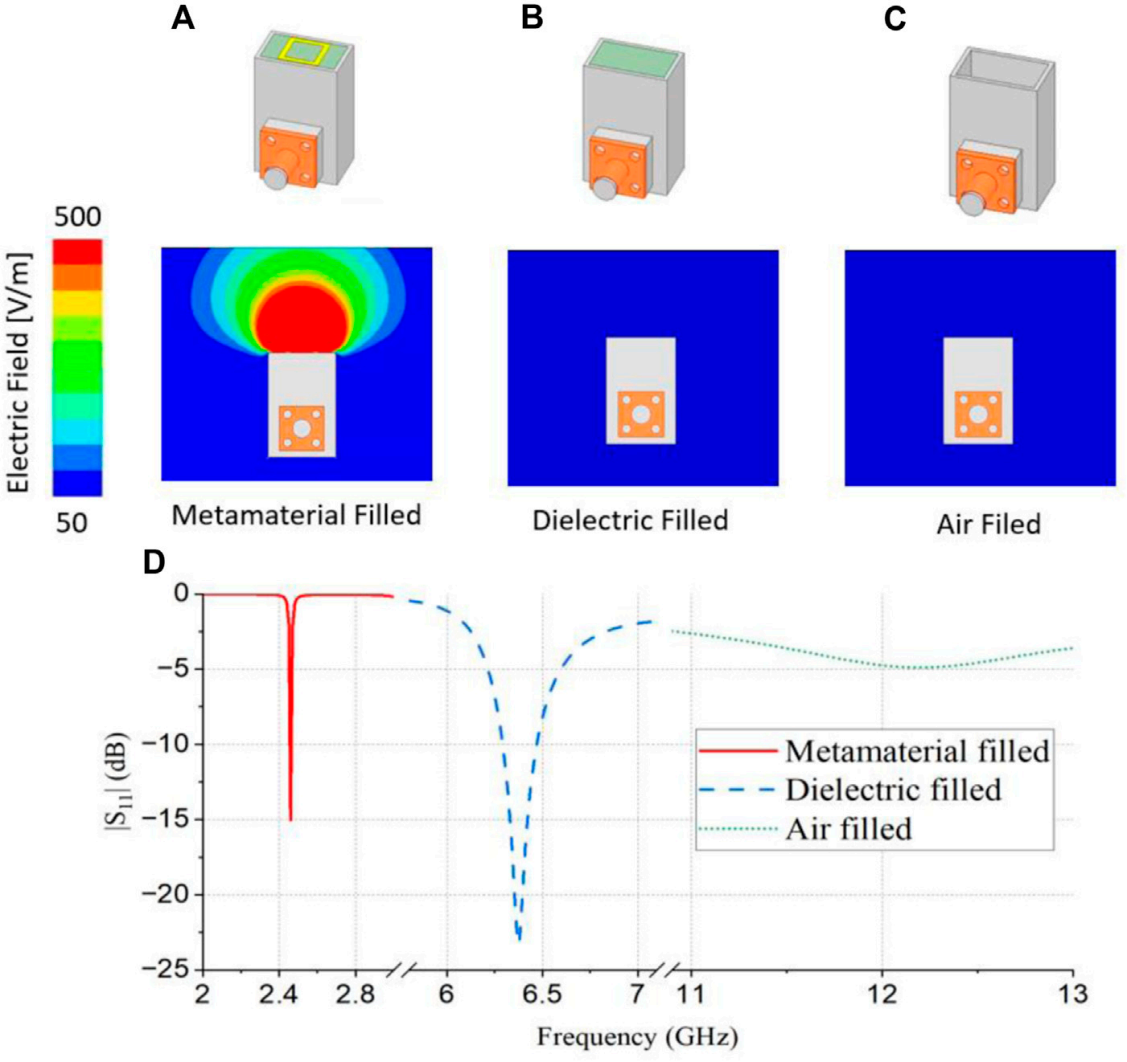
Electric field distribution at 2.45 GHz **(A)** metamaterial filled, **(B)** dielectric teflon-filled, and **(C)** air-filled waveguide aperture antenna, and **(D)** simulated reflection coefficients comparison between metamaterial-filled, dielectric teflon-filled, and air-filled waveguide aperture antenna of the same size.

Figure 3A show the overall, top, and cross-sectional views of the proposed compact metamaterial-filled waveguide aperture antenna. The stacked metamaterial was placed on an aluminum waveguide. It was fed by an SMA connector, and the metamaterial generated the ${TE}_{12}$ mode at 2.45 GHz. Figure 3B show the electric field distribution of waveguide that filled with metamaterial at 2.45 GHz and waveguide that filled with only dielectric at 5.58 GHz. We can see that without metamaterial ${TE}_{10}$ mode is dominate mode inside waveguide, but with metamaterial ${TE}_{12}$ mode is dominated. The proposed metamaterial-filled waveguide had a small aperture size of 10 mm × 17.4 mm (0.08λ × 0.14λ). A size comparison of the conventional and metamaterial-filled waveguide aperture antennas operating at 2.45 GHz is shown in Figure 4. Normally, the cutoff frequency of a rectangular waveguide is determined by Eq. 6, where c is the speed of light and W is the broadest wall of the rectangular waveguide. According to Eq. 6, the waveguide aperture antenna operating at 2.45 GHz should not be less than 61 mm. However, the proposed metamaterial-filled waveguide aperture antenna had the broadest compact wall size of 19.4 mm and operated well operating at 2.45 GHz, because of the metamaterials.

$$f_{c}=\frac{c}{2W}$$ (6)

Figures 4A–C show a comparison of the electric field distribution at 2.45 GHz for the metamaterial-filled antenna, dielectric (Teflon)-filled antenna, and air-filled antenna of the same size. The metamaterial-filled waveguide aperture antenna exhibited a strong upward electric field distribution and a weak backside electric field distribution in the near-field region. Thus, the proposed antenna heated only in the upward direction, ensuring user safety and stability within a very short time.

A dielectric- and air-filled waveguide aperture antenna of the same size as the metamaterial-filled waveguide aperture antenna exhibited a weak electric field both upward and backward because of the impedance mismatching at 2.45 GHz. Thus, the proposed metamaterial helped match the impedance and generated the ${TE}_{12}$ mode at 2.45 GHz.

Figure 4D shows a comparison of the simulated reflection coefficient values of the waveguide aperture antenna of the same size, filled with the proposed metamaterial, a dielectric (Teflon), and air. This indicates that with only air, which is a normal waveguide aperture antenna, a resonant frequency of approximately 12 GHz was achieved. When the dielectric (Teflon) was filled in the waveguide, the resonant frequency shifted to 6.4 GHz. However, when the waveguide was filled with the metamaterial, the resonant frequency shifted to 2.45 GHz. The proposed metamaterial significantly reduced the size and shifted the cut off frequency to the lower band of the waveguide-aperture antenna.

## 3 Results

**FIGURE 5:**
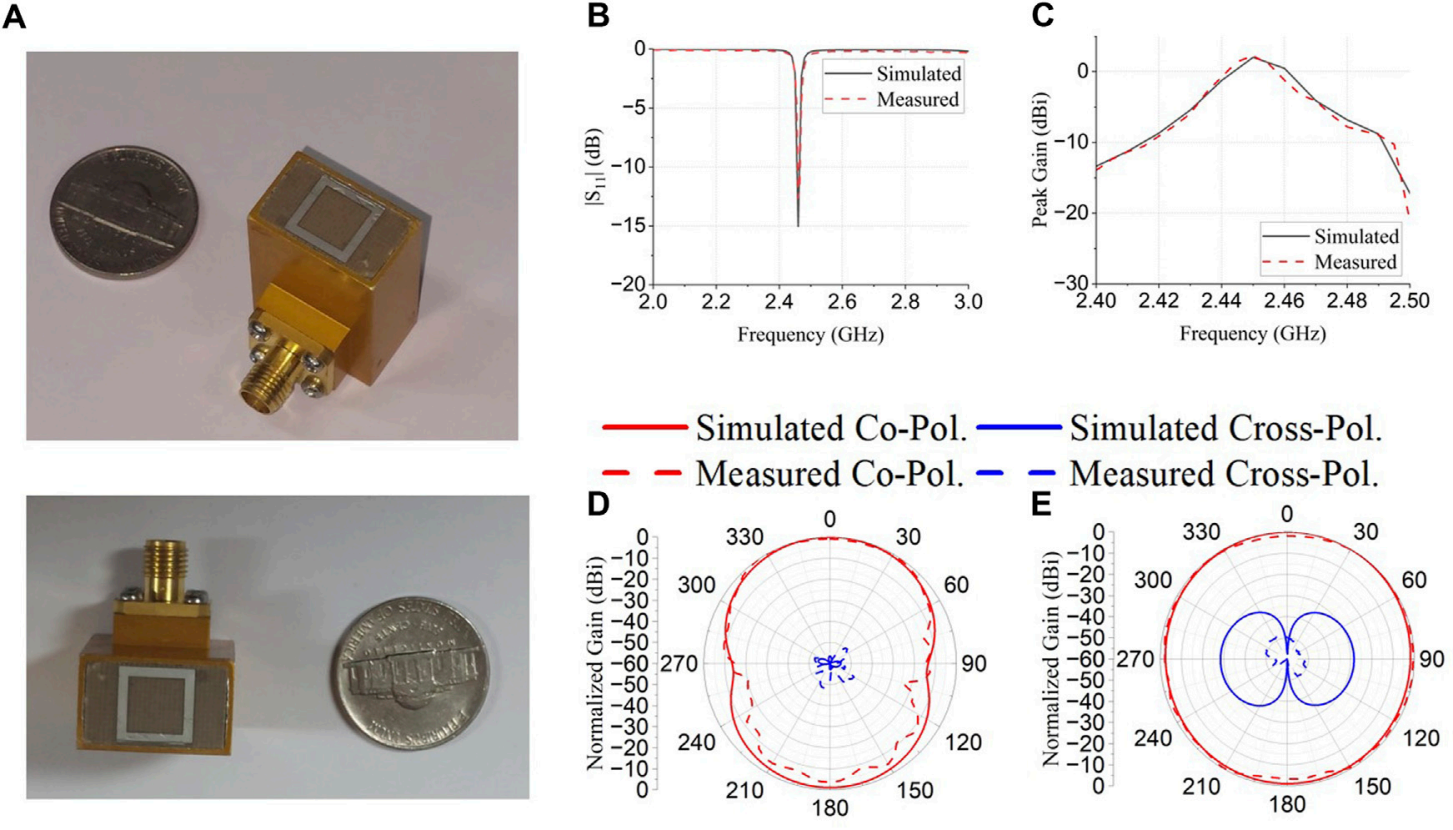
**(A)** Prototype of proposed metamaterial filled waveguide aperture antenna, simulated and measured **(B)** reflection coefficient, **(C)** peak gain, cross and co-polarization radiation pattern at **(D)** xz and **(E)** yz plane.

Figure 5 shows a prototype of the proposed compact metamaterial-filled antenna. The proposed antenna had a small aperture size of 10 mm × 17.4 mm for an electromagnetic field focused on a small area in the near-field region.

Figure 5 shows the simulated and measured (b) reflection coefficients and (c) peak gain. The simulated and measured results were consistent. The proposed antenna operated at 2.45 GHz with a measured peak gain of 2 dB. Figures 5D, E show the simulated and measured far-field co-polarization and cross-polarization radiation patterns of the proposed antenna in the xz and yz planes. This indicates good cross-polarization discrimination.

Because the proposed antenna operates in the near-field to increase the temperature, the distribution of the near-field electric field is important. If the near-field electric field does not propagate directly upward but instead radiates backward, the backside of the aperture antenna may also become heated, potentially causing damage to the cosmetic device user.

**FIGURE 6:**
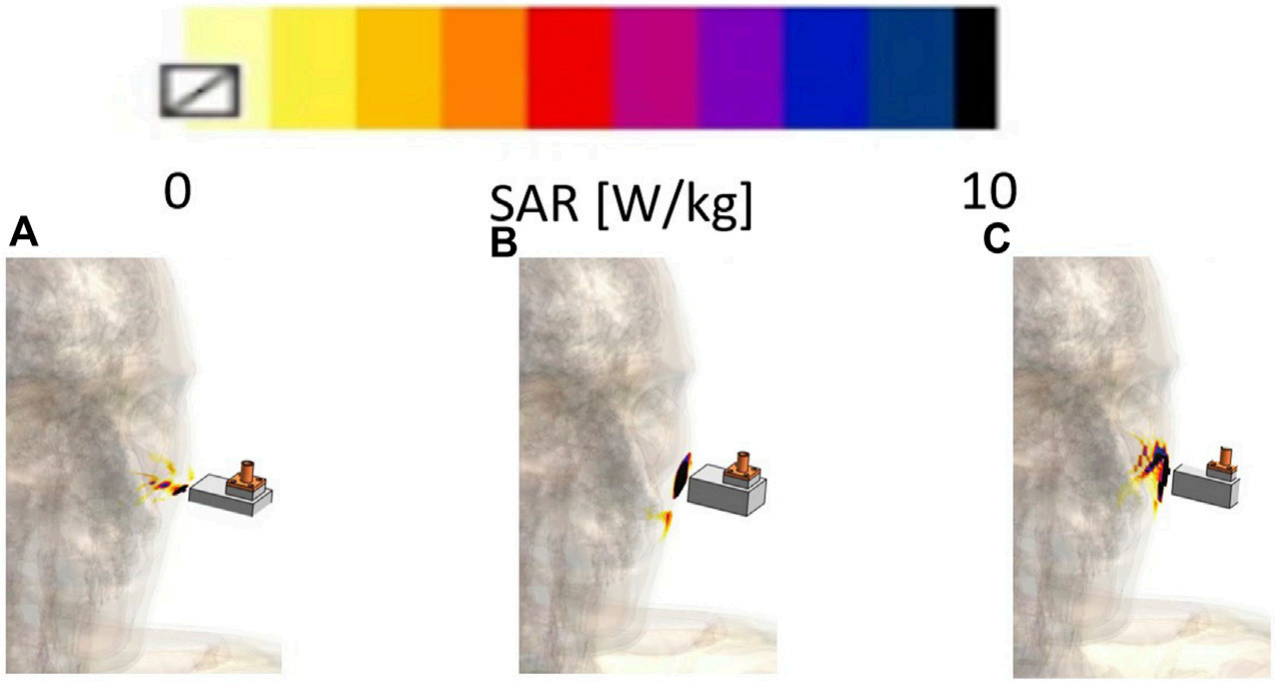
Simulated SAR with full 3D human body model Duke **(A)** xz, **(B)**, yz, and **(C)** xy plane.

Figure 6 displays the antenna toward the human face and the simulated SAR results for the (a) xz, (b) yz, and (c) xy plane cross-sectional views using the Sim4Life Duke model. The antenna aperture was positioned 4 mm above the Duke model (Gosseline and Neifield, 2014). When we analyzed the simulated SAR results in the xy cross-sectional view, we observed that the effective heating area on the surface was approximately 14 mm × 14 mm, which was a very narrow and focused area.

**FIGURE 7:**
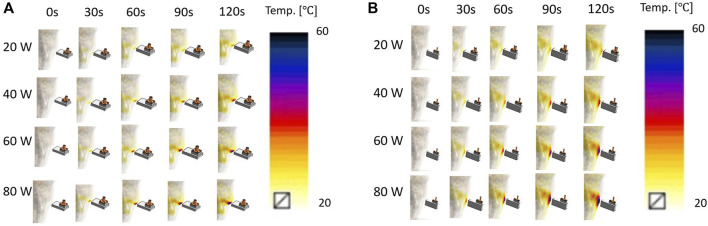
Simulated hyperthermia performance toward to human face **(A)** xz and **(B)** yz plane.

Figure 7 shows the simulated heating performance in the (a) xz and (b) yz plane cross-sectional views of a human face. The initial temperature of the Sim4Life Duke human body model was set to 20 °C in the simulation and the distance between the body model and antenna was 4 mm. The results demonstrate that the proposed antenna can focus heating on a specific area at a sufficient depth for skin lifting in a short time.

**FIGURE 8:**
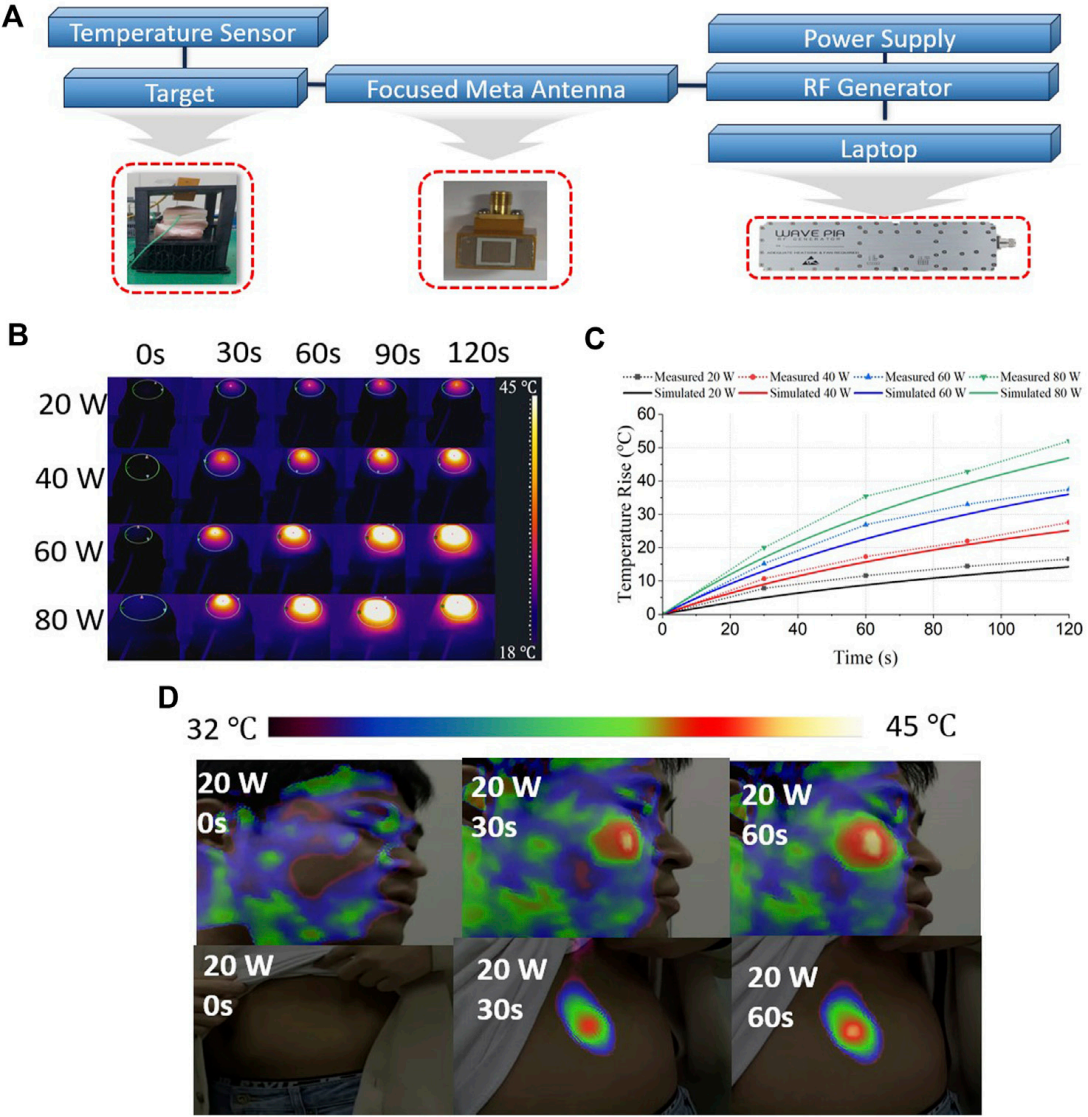
**(A)** Proposed RF hyperthermia system with compact metamaterial-filled waveguide aperture antenna, **(B)** measured hyperthermia performance by thermal camera at pork, **(C)** comparison of hyperthermia performance results between Sim4Life simulations and hyperthermia measurements in pork, and **(D)** measured RF hyperthermia performance toward to human face and valley.

Figure 8A shows the proposed RF hyperthermia system with a compact metamaterial-filled waveguide aperture antenna. An RF generator was connected to the proposed antenna to provide RF power at 2.45 GHz. A pork phantom was used to measure the heating performance, with the antenna positioned 4 mm above the pork phantom and a temperature sensor placed 4 mm below the pork skin surface. Examination the pork skin surface damage revealed that the effective area for microwave heating was 18 mm × 14 mm, showing a very narrow and focused area, similar to the Sim4Life-simulated SAR results.

Figure 8B shows the measured hyperthermia performance of the pork skin surface temperature captured by the thermal camera at different input RF power levels. In Figure 8C, we compare the simulated and measured temperatures at a depth of 4 mm below the skin surface.

The simulated temperature results were obtained from the Sim4Life Duke human model, specifically representing the temperature 4 mm below the skin surface of the face, whereas the measured results were obtained at a point 4 mm below the pork skin surface. The measured results indicate that the temperature could be raised to 35.4°C and 11.6°C within 60 s using 80 and 20 W of RF power, respectively.

Figure 8D shows the RF hyperthermia results for the human face and belly. The experimental subject was a 27-year-old male, whose antenna aperture was positioned 5–8 mm from the face and belly. The RF input power was 20 W at 2.45 GHz. Before RF hyperthermia, the face’s temperature was approximately 32°C. After 60 s of RF hyperthermia, the temperature increased to approximately 45°C at the target area. Prior to RF hyperthermia, the temperature of the abdomen was approximately 32°C. Following 60 s of RF hyperthermia, the temperature increased to approximately 43°C. The temperature rise results for pork and human faces were similar.

Table 1 presents a comparison between the proposed method and other methods. In the invasive method discussed in (Ganguly et al., 2023), low power was utilized for deep tumor hyperthermia; however, it required a significant amount of time to increase the temperature, which posed a risk to the patient. The noninvasive method discussed in (choi et al., 2014) also employed low power for shallow tumors and reduced the RF effective area by minimizing the radiator size using a meander line. However, a long time is required to increase the temperature. Kwon et al. (2019) and Rousseaux and Robson (2017) conducted clinical experimental research on RF hyperthermia cosmetic devices available in the commercial market. These studies applied relatively high power to the electrode to generate heat quickly and achieved a temperature increase of approximately 10°C. In contrast, our proposed approach increased the temperature by 11.6°C with only 20 W of input power within 60 s, focusing on the desired area. Therefore, the proposed method demonstrated the fastest and most efficient hyperthermia performance.

**TABLE 1:** Comparison between the proposed work and other works.

Works	Type	Frequency (GHz)	Method	Power (W)	Temperature rise	Target
[11]	Monopole	2.45	Invasive	2.5	10 ℃ (1500 s)	Deep tumor
[17]	Dipole	0.434	Non-Invasive	5	10 ℃ (3600 s)	Shallow tumor
[25]	Electrode	0.448	Non-invasive	200	11.1 ℃ (600 s)	Reduce subcutaneous fat
[26]	Electrode	0.001	Non-invasive	65	10 ℃ (180 s)	Skin tightening
Proposed	Metamaterial Filled Waveguide	2.45	Non-invasive	20/80	11.6/35.4 ℃ (60 s)	Skin tightening

## 4 Discussion

This study proposed a compact metamaterial-filled waveguide aperture antenna for noninvasive RF hyperthermia. The proposed antenna was designed to operate at 2.45 GHz to achieve cost efficiency. The compact size of the proposed waveguide aperture, enabled by the use of metamaterials, facilitated the focused RF energy delivery to a specific target area. The aperture size of the metamaterial-reduced waveguide was 98.8% smaller than that of the conventional waveguide.

Through experiments, we confirmed that the proposed antenna could withstand high power levels up to 100 W and raised the temperature by 11.6°C and 35.4°C with 20 and 80 W of input power, respectively, within 60 s. Furthermore, we conducted RF hyperthermia experiments on human faces and valley to verify its effectiveness. It can increase temperature rapidly with high power efficiency within an effective distance of 10 mm. The measurement results of the temperature increase demonstrate that the proposed antenna has the highest power and hyperthermia efficiency compared to other works and products in the skin-tightening hyperthermia market.

In the future, the proposed RF system will be combined with a cooler to prevent skin burns and maintain a cool temperature on the skin surface while the temperature below the skin surface remains high. The proposed RF hyperthermia systems will be utilized in the skin-tightening beauty device.

## Data Availability

The original contributions presented in the study are included in the article/Supplementary material, further inquiries can be directed to the corresponding authors.
